# Period prevalence of ocular inflammation and corneal allograft rejection within 30 days of COVID-19 vaccination

**DOI:** 10.1186/s12348-026-00629-3

**Published:** 2026-08-08

**Authors:** Sadegh Ghafarian, Sarah Pajek, Ruiyang Huang, Mona A. El Gendi, Jane Nguyen, Ann Pham, Andrea C. Santiago-Leon, Ellen H. Koo

**Affiliations:** 1 Department of Ophthalmology, School of Medicine, Farabi Eye Hospital, Tehran University of Medical Sciences, Tehran, Iran; 2 Department of Ophthalmology, Bascom Palmer Eye Institute, University of Miami Miller School of Medicine, 900 NW 17th St., Miami, FL 33136 USA

**Keywords:** COVID-19 vaccination, Ocular inflammation, Period prevalence, Uveitis, Herpetic keratitis, Corneal allograft rejection, Recurrence rate, Incidence

## Abstract

**Purpose:**

To determine the period prevalence of new or recurrent ocular inflammation, including corneal allograft rejection, occurring within 30 days after COVID-19 vaccination.

**Methods:**

This retrospective cohort study was conducted at a tertiary referral eye center. A systematic random sample of 4,638 patient charts (50% of eligible charts after removal of duplicate encounters identified by relevant ICD-10 codes) from December 1, 2020, to April 9, 2023, was reviewed using a computer-generated random start and a fixed sampling interval of 2. Patients were included if they developed new or recurrent ocular inflammation within 30 days of COVID-19 vaccination. Main outcome measures included the period prevalence of post-vaccination ocular inflammation, the proportion with new-onset inflammation, the proportion with recurrent inflammation among patients with prior inflammatory disease, and the frequency of corneal allograft rejection.

**Results:**

Among the 4,638 sampled patients with ocular inflammation-related ICD-10 codes, 77 met inclusion criteria, representing a period prevalence of 1.66% within 30 days of COVID-19 vaccination. The mean age was 58.0 years and 58.4% were male. Diagnoses included uveitis (*n* = 38), herpetic keratitis (*n* = 32), birdshot choroidopathy (*n* = 2), chorioretinitis (*n* = 2), pars planitis (*n* = 1), Staphylococcus-associated marginal keratitis (*n* = 1), and corneal allograft rejection (*n* = 1). Among patients with prior ocular inflammation, 0.65% experienced a recurrent event within 30 days of vaccination; among those without prior history, 1.01% had newly documented inflammation in the same window. The single episode of corneal allograft rejection occurred 13 days after the second dose of the Pfizer-BioNTech vaccine in a patient with prior Descemet stripping endothelial keratoplasty for Fuchs corneal dystrophy. Most cases responded to corticosteroid or antiviral therapy.

**Conclusion:**

Ocular inflammation within 30 days of COVID-19 vaccination was uncommon, and corneal allograft rejection was exceptionally rare in this large tertiary-center cohort. These findings support the continued use of COVID-19 vaccination in patients with prior ocular inflammatory disease or corneal transplantation, with appropriate post-vaccination monitoring.

## Key Message

**What is known:**

• Sporadic cases of ocular inflammation, including uveitis, herpetic keratitis, and rare corneal allograft rejection, have been reported following COVID-19 vaccination, raising concern among patients with prior inflammatory eye disease or corneal transplantation.

**New information from this study:**

• In a large tertiary-center cohort, ocular inflammation occurring within 30 days of COVID-19 vaccination was uncommon, with a low period prevalence of 1.66%.

• Both recurrence of pre-existing ocular inflammatory disease (0.65%) and new-onset ocular inflammation (1.01%) after vaccination were rare and generally responsive to standard medical therapy.

• Corneal allograft rejection following COVID-19 vaccination was exceptionally rare, with only a single documented case identified in this cohort.

## Introduction

During the global COVID-19 pandemic, the rapid development of vaccines based on novel mRNA technology offered an effective strategy for pandemic control, while simultaneously raising public concerns regarding potential adverse effects and long-term safety [1, 2]. As with other vaccines, SARS-CoV-2 vaccination of patients with transplants or with underlying autoimmune or inflammatory conditions had to be weighed against the risk of both post-vaccine inflammation and the risk of inflammation from COVID-19 itself. This included vaccinating patients with a history of uveitis, viral keratitis, or those who had received a corneal allograft transplant. Several case reports described ocular inflammation in such patients after SARS-CoV-2 vaccination [3–5].

Effective vaccinations include an intentional immune response for the development of new memory cells. For a variety of vaccines, this inflammatory response has been reported to cause unintentional ocular inflammation as well [6–8]. Although these ocular inflammatory events appear to be uncommon, they may be of particular concern in patients at increased risk of corneal allograft rejection or uveitis recurrence [3, 9].

In this descriptive population study, we examine the period prevalence of ocular inflammation and corneal allograft rejection occurring in patients who received the SARS-CoV-2 vaccination.

## Methods

**Fig. 1:**
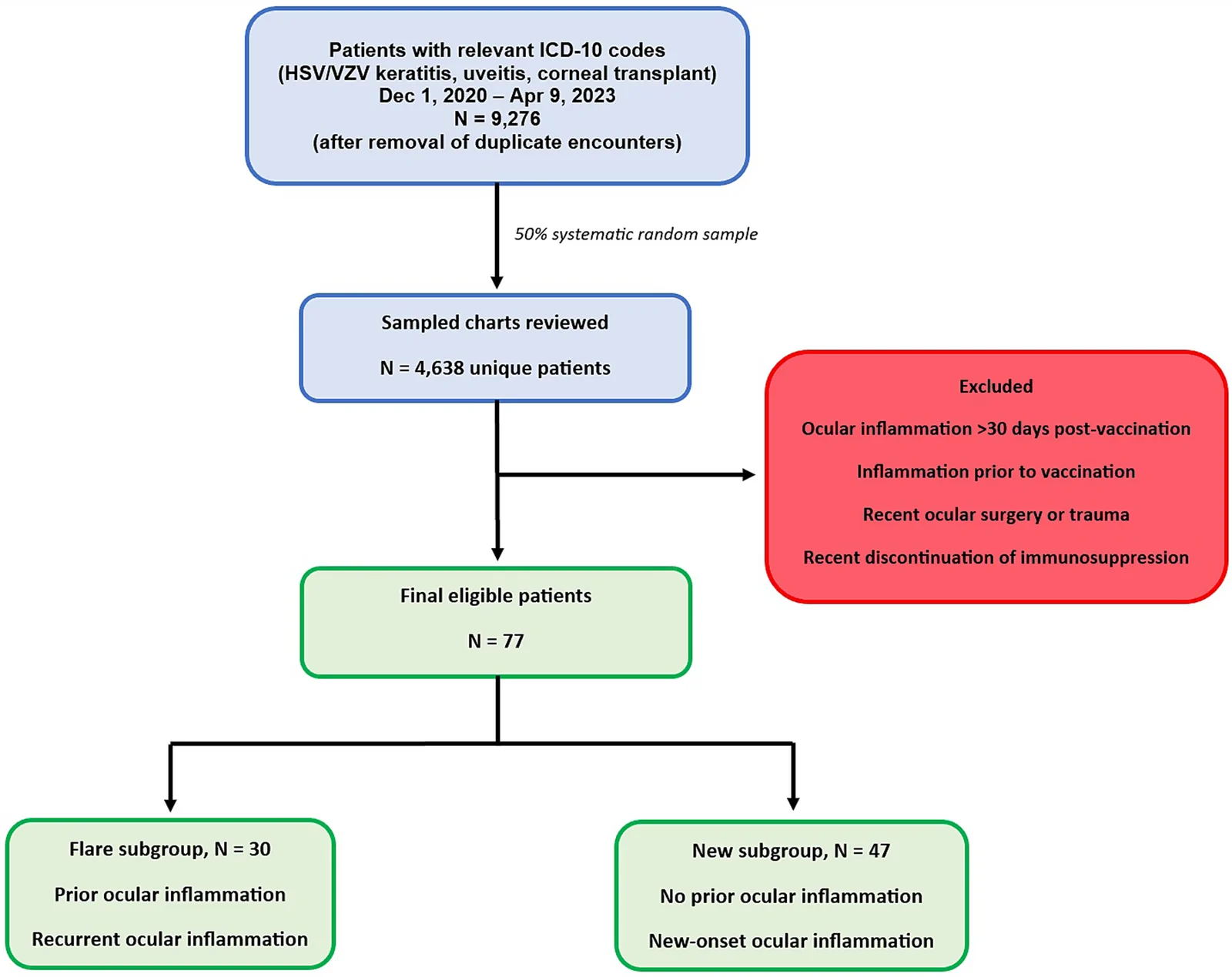
Flowchart of patient selection

This was a retrospective chart review using existing clinical data. The requirement for informed consent was waived by the University of Miami Institutional Review Board, as the study involved no prospective patient contact or intervention and posed minimal risk to participants. The study was approved by the University of Miami Institutional Review Board, was conducted in accordance with the principles of the Declaration of Helsinki, and was compliant with the Health Insurance Portability and Accountability Act. A retrospective chart review was conducted at the Bascom Palmer Eye Institute for the period from December 1, 2020, to April 9, 2023. During this interval, a total of 9,276 unique charts met the initial eligibility criteria on data extraction based on relevant ICD-10 diagnostic codes, after removal of duplicate patient encounters. Given the volume of records, full review of all eligible charts was not feasible; systematic random sampling was therefore used to select 50% of eligible charts for detailed review, yielding a final sample of 4,638 patients. A computer-generated random number was used to select the initial record, and every other chart was then selected (sampling interval = 2), giving each eligible patient an equal probability of inclusion. The eligible dataset was reviewed in the default order returned by the data extraction system, without sorting by date, provider, or diagnostic code (Fig. 1).

The included ICD-10 codes encompassed herpes simplex virus (HSV) keratitis (B00.52), varicella zoster virus (VZV) keratitis (B01.81), herpes zoster ophthalmicus (B02.30), keratitis (H16), iridocyclitis (H20), chorioretinal inflammation (H30), chorioretinal disorders in diseases classified elsewhere (H32), disorders of the globe (H44), and complications of corneal transplantation (T86.84).

Patients aged 18 years or older were included if they were newly diagnosed or had a reactivation of uveitis, infectious keratitis, or corneal allograft rejection in the immediate post-vaccination period, defined as within 30 days after COVID-19 vaccination. Patients were excluded if the ocular inflammatory event began prior to COVID-19 vaccination, occurred more than 30 days after vaccination, if an immunosuppressive medication had been discontinued at the prior visit, or if ocular surgery or trauma had occurred within the preceding 30 days. Ultimately, 77 patients met the criteria and were included in the final analysis (Fig. 1). Additional data for these patients was recorded, including demographics, smoking status, autoimmune diagnoses, cancer diagnoses, ocular comorbidities, vaccine brand, treatment plan and days to inflammation resolution. Resolution was defined on the basis of clinician documentation of symptomatic and clinical resolution at follow-up, including absence of ciliary injection, active anterior chamber cells, keratic precipitates (KPs), and corneal edema where applicable based on the presenting diagnosis. Time to resolution was calculated from the date of diagnosis. Patients lost to follow-up were excluded from resolution analyses.

## Data analysis

Patients with new ocular inflammation were analyzed as two separate subgroups: those with history of ocular inflammation that was quiescent prior to vaccination (“Flare”) and those with no prior known history of ocular inflammation (“New”) (Fig. 1). Corneal allograft rejection was analyzed in the Flare subgroup. Data was analyzed using SPSS Statistics, Version 25.0 software (IBM Corp., Armonk, NY, USA). Figures and tables were created using Microsoft Excel version 16.61.1.

## Results

Among the 4,638 sampled patients with ocular inflammation-related ICD-10 codes, 77 (1.66%) had ocular inflammation documented within 30 days of COVID-19 vaccination. The mean age was 57.64 years (SD, 16.12; range, 18–89 years), and 45 patients (58.4%) were male. (Table 1). Age was similar for both subgroups, but the new subgroup had a higher proportion of male patients (32/47, 68.1%). Most patients were White (66/77, 85.7%), with about half of these being White Hispanic (36/77, 46.8%). Most patients also identified as “never smokers” (52/77,67.5%) and 16 (20.8%) had a history of diabetes mellitus (Table 1).

**Table 1:** Demographics

	Overall	Flare subgroup	New subgroup
N (%)	77 (100%)	30 (38.96%)	47 (61.04%)
Age (years)	57.64 (SD: 16.12)	56.84 (SD: 18.67)	58.15 (SD: 14.45)
Sex	45 male (58.44%) 33 female (42.86%)	13 male (43.33%) 17 female (56.67%)	32 male (68.09%) 16 female (34.04%)
Race	66 White (85.71%) 7 Black (9.091%) 3 Asian (3.896%) 1 unknown (1.299%)	24 White (80.00%) 4 Black (13.33%) 1 Asian (3.33%) 1 unknown (3.33%)	42 White (89.36%) 3 Black (6.383%) 2 Asian (4.255%)
Ethnicity	36 Hispanic (46.75%)	12 Hispanic (40.00%)	24 Hispanic (51.06%)
Smoking status	4 current (5.195%) 21 former (27.27%) 52 never (67.53%)	1 current (3.33%) 9 former (30.00%) 20 never (66.67%)	3 current (6.383%) 12 former (25.53%) 32 never (68.09%)
Diabetes	16 (20.78%)	5 (16.67%)	11 (23.40%)
At least one ocular Comorbidities	35 (45.5%)	21 (70.00%)	14 (29.8%)
Medical condition affecting immune status	24 (31.37%)	17 (56.67%)	7 (14.89%)

A total of 35 patients (45.5%) had at least one chronic ocular comorbidity, including 21 patients (70.0%) in the Flare subgroup and 14 patients (29.8%) in the New subgroup (Table 1). In the Flare subgroup, ocular comorbidities included glaucoma in 12 patients (40.0%), cystoid macular edema in 4 (13.3%), Fuchs corneal dystrophy in 3 (10.0%), historical retinal detachment in 2 (6.7%), non-proliferative diabetic retinopathy in 2 (6.7%), corneal edema in 2 (6.7%), macular degeneration in 1 (3.3%), retinal vasculitis in 1 (3.3%), and non-arteritic anterior ischemic optic neuropathy (NAION) in 1 (3.3%). In the New subgroup, ocular comorbidities included glaucoma in 8 patients (17.0%), non-proliferative diabetic retinopathy in 4 (8.5%), cystoid macular edema in 3 (6.4%), macular degeneration in 1 (2.1%) and keratoconus in 1 (2.1%).

Regarding medical conditions affecting the immune system, 24 patients (31.4%) had such conditions, including 17 (56.7%) in the Flare subgroup and 7 (14.9%) in the New subgroup (Table 1). In the Flare subgroup, immunocompromising conditions included rheumatoid arthritis in 6 patients (20.0%), birdshot choroidopathy in 2 (6.7%), ankylosing spondylitis in 2 (6.7%), multiple sclerosis in 1 (3.3%), Vogt-Koyanagi-Harad in 1 (3.3%), dermatomyositis in 1 (3.3%), Hashimoto’s thyroiditis in 1 (3.3%), discoid lupus in 1 (3.3%), recent postpartum status in 1 (3.3%), Waldenström macroglobulinemia in 1 (3.3%). Of these, 10 (33.3%) were taking immunosuppressant medications and 5 were HLA-B27 positive (16.7%). In the New subgroup, immunocompromising conditions included psoriasis in 2 patients (4.3%), concurrent use of oral prednisone in 2 (4.3%), ankylosing spondylitis in 1 (2.1%), uveal melanoma undergoing treatment in 1 (2.1%), and metastatic lung adenocarcinoma undergoing chemotherapy in 1 (2.1%).

Of 77 (1.66%) who had ocular inflammation documented within 30 days of COVID-19 vaccination, 30 (39.0%) occurred in patients with a distant history of ocular inflammation or corneal allograft transplantation, representing 0.65% of the sampled cohort (Table 2). 19 (63.3%) were cases of recurrent uveitis, including 12 (15.6%) cases of anterior uveitis, 3 (3.9%) of intermediate uveitis, 2 (2.6%) of posterior uveitis, and 2 (2.6%) of panuveitis. 7 (23.3%) were viral, including 4 (5.2%) related to VZV and 3 (3.9%) to HSV. Other flare cases included 2 (2.6%) of birdshot choroidopathy and 1 (1.3%) of Staphylococcus-associated marginal keratitis. There was also 1 (1.3%) case of corneal allograft rejection. 47 (61.0%) cases occurred in patients without prior history of ocular inflammation, representing 1.01% of the sampled cohort (Table 2). This included 22 (28.6%) cases of anterior uveitis, 16 (20.8%) cases related to HSV, and 9 (11.7%) cases related to VZV.

**Table 2:** Cumulative incidence by subgroup

	Total *n* (%)	Cumulative incidence
All	77 (100%)	1.66%
Flare subgroup	30 (38.96%)	0.65%
Anterior uveitis	12 (15.58%)	0.26%
Intermediate uveitis	3 (3.896%)	0.06%
Posterior uveitis	2 (2.597%)	0.04%
Panuveitis	2 (2.597%)	0.04%
VZV-related	4 (5.195%)	0.09%
HSV-related	3 (3.896%)	0.06%
Birdshot choroidopathy	2 (2.597%)	0.04%
Staphylococcus- associated marginal keratitis	1 (1.299%)	0.02%
Corneal allograft rejection	1 (1.299%)	0.02%
New subgroup	47 (61.04%)	1.01%
Anterior uveitis	22 (28.57%)	0.47%
HSV-related	16 (20.78%)	0.34%
VZV-related	9 (11.69%)	0.19%

The timing of ocular inflammatory events following COVID-19 vaccination was as follows: most cases (45, 58.4%) occurred after the first dose (15 [50.0%] in the Flare subgroup, 30 [63.8%] in the New subgroup; Table 3), 26 cases (33.8%) occurred after the second dose (10 [33.3%] Flare, 16 [34.0%] New), and 6 cases (7.8%) occurred after the third dose (5 [16.7%] Flare, 1 [2.1%] New). Vaccine brands received prior to the ocular inflammation diagnosis included 53 (68.8%) Pfizer-BioNTech, 21 (27.3%) Moderna, and 3 (3.9%) Johnson & Johnson (Table 4). Mean time from vaccination to diagnosis was 14.49 (SD: 8.632) days (Table 5). All patients achieved symptom resolution except the 1 corneal allograft rejection case (1.3%). Time to symptom resolution was 59.17 (SD: 49.44) days for the Flare subgroup and 26.68 (SD: 29.95) days for the New subgroup (Table 5). Patients who were lost to follow-up were excluded from resolution analysis: 5 (16.7%) in the Flare subgroup and 7 (14.9%) in the New subgroup.

**Table 3:** Related vaccine dose by diagnosis

	Dose 1 *n* (%)	Dose 2 *n* (%)	Dose 3 *n* (%)
All	45 (58.44%)	26 (33.77%)	6 (7.79%)
Flare subgroup	15 (50.00%)	10 (33.33%)	5 (16.67%)
Anterior uveitis	5 (41.67%)	6 (50.00%)	1 (8.333%)
Intermediate uveitis	1 (33.33%)	2 (66.67%)	-
Posterior uveitis	1 (50.00%)	-	1 (50.00%)
Panuveitis	1 (50.00%)	-	1 (50.00%)
VZV-related	4 (100%)	-	-
HSV-related	2 (66.67%)	-	1 (33.33%)
Birdshot choroidopathy	1 (50.00%)	-	1 (50.00%)
Staphylococcus- associated marginal keratitis	-	1 (100%)	-
Corneal allograft rejection	-	1 (100%)	-
New subgroup	30 (63.83%)	16 (34.05%)	1 (2.128%)
Anterior uveitis	17 (77.27%)	5 (10.64%)	
HSV-related	11 (68.75%)	4 (25.00%)	1 (6.250%)
VZV-related	2 (22.22%)	7 (77.78%)	

**Table 4:** Related vaccine brand by diagnosis

	Pfizer *n* (%)	Moderna *n* (%)	Johnson & Johnson *n* (%)
All	53 (68.83%)	21 (27.27%)	3 (3.90%)
Flare subgroup	22 (73.33%)	8 (26.67%)	-
Anterior uveitis	9 (75.00%)	3 (25.00%)	-
Intermediate uveitis	2 (66.67%)	1 (33.33%)	-
Posterior uveitis	1 (50.00%)	1 (50.00%)	-
Panuveitis	2 (100%)	-	-
VZV-related	2 (50.00%)	2 (50.00%)	-
HSV-related	2 (66.67%)	1(33.33%)	-
Birdshot choroidopathy	2 (100%)	-	-
Staphylococcus- associated marginal keratitis	1 (100%)	-	-
Corneal allograft rejection	1 (100%)	-	-
New subgroup	31 (65.96%)	13 (27.66%)	3 (6.383%)
Anterior uveitis	16 (72.73%)	4 (18.18%)	2 (9.091%)
HSV-related	9 (56.25%)	7 (43.75%)	-
VZV-related	6 (66.67%)	2 (22.22%)	1 (11.11%)

**Table 5:** Days to diagnosis and to clinical resolution

	Mean interval from vaccine to diagnosis (days)	Mean time to resolution (days)
All	14.49 (SD: 8.632)	38.86 (SD: 41.22)*
Flare subgroup	13.6 (SD: 9.137)	59.17 (SD: 49.44)*
Anterior uveitis	12.67 (SD: 9.306)	61.00 (SD: 48.46)*
Intermediate uveitis	7.67 (SD: 5.03)	108.50 (SD: 27.58)*
Posterior uveitis	15.50 (SD: 2.12)	40.00 (SD: 14.14)
Panuveitis	21.00 (SD: 12.73)	93.00 (SD: 93.34)
VZV-related	16.25 (SD: 13.55)	52.75 (SD: 54.84)
HSV-related	14.33 (SD: 11.02)	2*
Birdshot choroidopathy	11.50 (SD: 13.44)	43*
Staphylococcus- associated marginal keratitis	16	10
Corneal allograft rejection	13	not applicable
New subgroup	15.06 (SD: 8.34)	26.68 (SD: 29.95)*
Anterior uveitis	13.95 (SD: 8.88)	25.26 (SD: 29.65)*
HSV-related	15.19 (SD: 7.90)	25.13 (SD: 32.27)*
VZV-related	17.56 (SD: 8.11)	35.00 (SD: 28.57)*

*Excludes patients that were lost to follow up

Treatment of uveitis in the Flare subgroup included ophthalmic prednisolone acetate 1% for 15 patients (79.0%), ophthalmic cyclopentolate for 5 (26.3%), oral prednisone for 2 (10.5%), ophthalmic difluprednate 0.05% for 2 (10.5%), and ophthalmic dexamethasone/neomycin/polymyxin for 1 (5.3%). In the New subgroup, uveitis treatments included ophthalmic prednisolone acetate 1% for 22 patients (100%), ophthalmic cyclopentolate for 12 (54.5%), oral valacyclovir for 4 (18.2%), ophthalmic atropine for 3 (13.6%), and ophthalmic ketorolac tromethamine for 1 (4.5%).

In the Flare subgroup, all patients with viral diagnoses (HSV and VZV keratitis and keratouveitis, consistent with the ICD-10 codes used for patient identification) were treated with oral valacyclovir, with adjunctive topical corticosteroids used in 5 patients (71.4%). In the New subgroup, oral valacyclovir was used in 24 patients (96.0%), with additional therapies including topical corticosteroids (36.0%), cycloplegics (24.0%), and topical antivirals (16.0%). One patient receiving concurrent chemotherapy required intravenous foscarnet and ganciclovir.

Of the 2 Flare cases of birdshot choroidopathy, 1 (50.0%) did not receive additional treatment and 1 (50.0%) received a dexamethasone implant. The case of Staphylococcus-associated marginal keratitis in the Flare subgroup was treated with oral doxycycline, ophthalmic polymyxin B sulfate/trimethoprim, and ophthalmic fluorometholone.

The case of corneal graft rejection was diagnosed 13 days after the patient’s second dose of Pfizer-BioNTech COVID-19 vaccination (Tables 3 and 4). It occurred 570 days after the initial descemet’s stripping endothelial keratoplasty (DSEK) procedure for the treatment of Fuchs corneal dystrophy. The rejection was treated with prednisolone without improvement and subsequent allograft transplantation was required.

## Discussion

More than five years after the COVID-19 pandemic, which has resulted in hundreds of millions of confirmed infections and millions of deaths worldwide [10], there is now sufficient clinical experience to critically evaluate not only the effectiveness of vaccination strategies but also their potential short- and intermediate-term complications.

Uveitis is among the most commonly reported ophthalmic adverse events following COVID-19 vaccination, with more than 70 published case reports and case series to date [11, 12]. Consequently, patients with a history of uveitis, keratoplasty, or other immune-mediated ocular conditions may be particularly concerned about post-vaccination inflammation, although evidence linking COVID-19 vaccination to an increased risk of uveitis flare remains controversial [1, 13–16]. A large analysis of vaccine adverse event reports identified 1,094 cases of vaccine-associated uveitis from 40 countries, demonstrating a low crude reporting rate and suggesting a possible temporal association between reported uveitis events and COVID-19 vaccination, without evidence of a significant safety signal [4].

In the current study, the period prevalence of ocular inflammation occurring within 30 days of COVID-19 vaccination was 1.66% among patients with ocular inflammation-related ICD-10 codes, indicating that such events are very uncommon. However, this figure does not represent the incidence or prevalence of post-vaccination ocular inflammation in the broader vaccinated population. Because the denominator was restricted to patients with inflammatory diagnoses at a tertiary referral center, the proportion of all vaccinated individuals who develop such events is likely considerably lower. Importantly, a temporal association between vaccination and an inflammatory event does not necessarily establish causality [12, 16]. Recurrent uveitis occurred in 19 of 4,638 patients (0.41%), which is higher than the estimated recurrence rate in the general population of approximately 0.02–0.05% per year [17–19]; however, this difference likely reflects the enrichment of patients with pre-existing ocular inflammatory disease in the study cohort rather than a causal effect of vaccination.

The pathogenesis of uveitis following COVID-19 vaccination remains incompletely understood and is likely multifactorial. Proposed mechanisms include molecular mimicry between vaccine antigens and uveal self-peptides, delayed-type hypersensitivity reactions, and immune responses directed against vaccine adjuvants, all of which may disrupt ocular immune privilege [16, 20].

Vaccination-induced increases in vascular permeability may further facilitate immune complex deposition within the uvea and iris, triggering localized inflammation [21]. mRNA vaccines may contribute to ocular inflammation through several immunological mechanisms. Certain vaccine components, such as polyethylene glycol used in mRNA delivery systems, can activate complement pathways [22–24], while the mRNA platform itself stimulates innate immunity via endosomal and cytoplasmic nucleic acid receptors, resulting in activation of proinflammatory signaling cascades that may be amplified in individuals with underlying autoimmune susceptibility [25–27]. In addition, the RNA component may directly activate type I interferon responses, potentially inducing retinal pigment epithelium cell injury and providing an additional mechanistic pathway for post-vaccination ocular inflammation [28–30].

The second most common conditions observed in our study were viral manifestations (HSV and VZV), affecting 7 patients (9.1%) in the Flare subgroup and 25 patients (16.1%) in the New subgroup. The recurrence rates for HSV and VZV were 0.4% and 0.3%, respectively, which exceed the reported recurrence rate of HSV keratitis in the USA, estimated at 15.6 per 100,000 person-years [31]. Previous studies have indicated that both new onset and recurrent HSV keratitis may be more likely following COVID-19 vaccination [32, 33]. An earlier report on infectious keratitis post-COVID-19 vaccination documented a mean interval of 12.3 ± 10.3 days between vaccination and symptom onset [34], comparable to the interval observed in our cohort. Vaccines are hypothesized to trigger viral reactivation via T-cell-mediated immune activation and upregulation of proinflammatory cytokines [35]. The cases presented here are consistent with prior reports, suggesting that COVID-19 vaccination may be temporally associated with herpesvirus reactivation even after prolonged periods of quiescence [36, 37]. Accordingly, prophylactic antiviral therapy may be considered in selected patients with a history of herpetic keratitis who are deemed to be at high risk of recurrence following vaccination [38].

In addition, we report the second known case of post-vaccination Staphylococcus-associated marginal keratitis [39]. This case involved a 60-year-old female who experienced recurrence 16 days after her second dose of the Pfizer-BioNTech vaccine and achieved symptom resolution within 10 days following appropriate treatment. We also identified two cases of birdshot choroidopathy exacerbation after vaccination, both occurring in females in their 80s. One patient achieved symptom resolution in 43 days, while the other was lost to follow-up. To our knowledge, only one prior case has been reported, a 54-year-old female who developed exacerbation six weeks after receiving the Sputnik COVID-19 vaccine, with no reported time to resolution [40].

In this cohort, most post-vaccination inflammatory events occurred following mRNA-based vaccines (Pfizer-BioNTech and Moderna), with relatively few cases observed after the Johnson & Johnson vaccine. These distributions are consistent with prior reports and likely reflect the predominant use of mRNA vaccines during the study period [4]. Inflammatory events were most frequently observed after the first vaccine dose, with fewer cases after the second and only a small proportion after subsequent doses. These dose distributions are consistent with two large population-based studies reporting a higher frequency of inflammatory events after the initial vaccine dose [4, 41]. However, some reports have described a higher frequency of uveitis following the second or subsequent doses of COVID-19 vaccination, underscoring variability in mechanism and individual immune responses [16, 27, 42]. The time to uveitis diagnosis after COVID-19 vaccination has previously been reported to average 8.0 ± 8.6 days after vaccination [11]. In our cohort, the time to diagnosis varied by uveitis subtype, with the earliest onset observed in intermediate uveitis flares (7.67 ± 5.03 days) and the latest onset in panuveitis (21.00 ± 12.73 days) (Table 5).

Given reports of corneal allograft rejection occurring within several weeks of COVID-19 vaccination, it is important to assess whether vaccination may be associated with an increased risk of graft rejection [43–45]. An analysis of the Centers for Disease Control(CDC) Vaccine Adverse Event Reporting(VAER) System identified 46 patients (55 eyes) with reported corneal graft rejection following COVID-19 vaccination, predominantly after mRNA vaccines, and suggested a possible temporal association, with earlier onset observed after the second vaccine dose and in patients with a history of endothelial keratoplasty [46]. In our cohort, only a single case of corneal allograft rejection was identified. The patient was a 67-year-old woman who had undergone Descemet stripping endothelial keratoplasty (DSEK) for Fuchs corneal dystrophy 570 days prior to presentation, with rejection occurring 13 days after the second dose of the Pfizer-BioNTech vaccine. Despite treatment with topical prednisolone acetate 1%, the rejection did not resolve and ultimately required repeat keratoplasty. Accordingly, cornea specialists should closely monitor patients with a history of keratoplasty after COVID-19 vaccination and counsel them to promptly report symptoms such as photophobia, ocular pain, or reduced vision.

In our study, most patients with uveitis responded favorably to corticosteroid therapy, with 98.7% achieving symptom resolution over a mean duration of 38.86 ± 41.22 days, a response pattern consistent with an immune-mediated mechanism underlying post-vaccination uveitis. Beyond their therapeutic role, concomitant use of systemic glucocorticoids at the time of COVID-19 vaccination has been associated with a dose-dependent reduction in the risk of post-vaccination uveitis relapse [16, 47]. In the setting of post-vaccination corneal allograft rejection, early initiation of topical corticosteroid therapy has been reported to achieve complete reversal of rejection episodes [43]. Importantly, short-term corticosteroid use in the peri-vaccination period has been shown to be safe, reducing vaccine-related inflammatory adverse effects without impairing vaccine-elicited serologic antibody responses [48].

In the current study, inflammatory events following COVID-19 vaccination were rare and responded favorably to standard medical therapy. These findings should be interpreted in the context of the risks associated with COVID-19 infection itself. SARS-CoV-2 infection can result in a range of systemic and ocular manifestations [49, 50] and has been shown to significantly increase the risk of uveitis recurrence, whereas this risk appears to be reduced in individuals who contract COVID-19 after prior vaccination [51]. SARS-CoV-2 proteins have been detected in tear samples up to three months after the initial infection in reported cases of post-infectious viral keratouveitis [52], and viral RNA has also been demonstrated in the aqueous humor of asymptomatic individuals. These findings raise the possibility that SARS-CoV-2 may persist within immunoprivileged ocular compartments after the acute phase of infection [53], underscoring the importance of preventing infection in the first place.

This study has several limitations. First, its retrospective design and reliance on chart review introduce potential inaccuracies and incomplete documentation. Reliance on self-reported vaccination history in some cases could introduce recall bias, and that the precise proportion of cases verified exclusively by patient report could not be determined from the available retrospective data. However, where possible, vaccine date, brand, and dose number were verified through documented clinical records.

Second, because patients were identified using ICD-10 codes for ocular inflammation, the denominator represents an enriched inflammatory cohort rather than all vaccinated individuals; therefore, the calculated proportions do not reflect population-level incidence and this study does not assess vaccine safety at the population level. Third, the date of diagnosis may not precisely correspond to the true onset of inflammation or graft rejection, and in some cases, symptoms may have preceded vaccination but were diagnosed afterward. Finally, the observational design precludes inference of causality, and temporal association alone cannot establish a direct pathogenic relationship between vaccination and ocular inflammation. This is particularly relevant to the New subgroup, in which diagnoses such as HSV/VZV keratitis and anterior uveitis can occur spontaneously and may be entirely unrelated to vaccination.

In conclusion, ocular inflammation within 30 days of COVID-19 vaccination was uncommon in this large tertiary-center cohort, and corneal allograft rejection was exceptionally rare. These findings support the continued use of COVID-19 vaccination in patients with prior ocular inflammatory disease or corneal transplantation, with appropriate post-vaccination monitoring. Future prospective, population-based studies are needed to better characterize the true incidence of post-vaccination ocular events, identify modifiable risk factors, and establish whether a causal relationship exists between COVID-19 vaccination and specific ocular inflammatory conditions.

## Data Availability

Data will be made available upon reasonable request.
